# Opposing Activity Changes in AMP Deaminase and AMP-Activated Protein Kinase in the Hibernating Ground Squirrel

**DOI:** 10.1371/journal.pone.0123509

**Published:** 2015-04-09

**Authors:** Miguel A. Lanaspa, L. Elaine Epperson, Nanxing Li, Christina Cicerchi, Gabriela E. Garcia, Carlos A. Roncal-Jimenez, Jessica Trostel, Swati Jain, Colin T. Mant, Christopher J. Rivard, Takuji Ishimoto, Michiko Shimada, Laura Gabriela Sanchez-Lozada, Takahiko Nakagawa, Alkesh Jani, Peter Stenvinkel, Sandra L. Martin, Richard J. Johnson

**Affiliations:** 1 Division of Renal Diseases and Hypertension, University of Colorado Denver, Aurora, CO, 80045, United States of America; 2 Department of Cell and Developmental Biology, Aurora, CO, 80045, United States of America; 3 Department of Biochemistry and Molecular Genetics, Aurora, CO, 80045, United States of America; 4 Laboratory of Renal Physiopathology and Nephrology Dept, INC Ignacio Chavez, Mexico City, Mexico; 5 Division of Renal Medicine, Department of Clinical Intervention and Technology, Karolinska Institutet, Stockholm, Sweden; 6 Division of Nephrology, Eastern Colorado Health System, Department of Veteran Affairs, Denver, CO, United States of America; University of Santiago de Compostela School of Medicine - CIMUS, SPAIN

## Abstract

Hibernating animals develop fatty liver when active in summertime and undergo a switch to a fat oxidation state in the winter. We hypothesized that this switch might be determined by AMP and the dominance of opposing effects: metabolism through AMP deaminase (AMPD2) (summer) and activation of AMP-activated protein kinase (AMPK) (winter). Liver samples were obtained from 13-lined ground squirrels at different times during the year, including summer and multiples stages of winter hibernation, and fat synthesis and β-fatty acid oxidation were evaluated. Changes in fat metabolism were correlated with changes in AMPD2 activity and intrahepatic uric acid (downstream product of AMPD2), as well as changes in AMPK and intrahepatic β-hydroxybutyrate (a marker of fat oxidation). Hepatic fat accumulation occurred during the summer with relatively increased enzymes associated with fat synthesis (FAS, ACL and ACC) and decreased enoyl CoA hydratase (ECH1) and carnitine palmitoyltransferase 1A (CPT1A), rate limiting enzymes of fat oxidation. In summer, AMPD2 activity and intrahepatic uric acid levels were high and hepatic AMPK activity was low. In contrast, the active phosphorylated form of AMPK and β-hydroxybutyrate both increased during winter hibernation. Therefore, changes in AMPD2 and AMPK activity were paralleled with changes in fat synthesis and fat oxidation rates during the summer-winter cycle. These data illuminate the opposing forces of metabolism of AMP by AMPD2 and its availability to activate AMPK as a switch that governs fat metabolism in the liver of hibernating ground squirrel.

## Introduction

Body weight is tightly regulated in most species. For example, Keesey et al have shown that rats return to their baseline weight after either force feeding or force fasting [1]. Migrating songbirds that fast during the summer regain their weight during the recovery phase [2]. In both situations the animals return to their baseline weight for their age and the time of the year. Many species also gain weight in preparation for periods of food shortage. For example, the Emperor penguin (*Aptenodytes forsteri*) doubles its body weight in fat to survive for up to 4 months on the Antarctic ice during brooding [3]. Hibernating mammals also provide excellent examples of animals that gain large amounts of fat and then lose it while fasting throughout winter [4].

Ground squirrels and other sciurid rodent hibernators actively accumulate fat in the fall, and then live off their fat stores throughout the many months of winter hibernation [5,6]. In the fall, weight gain may be enhanced by reduced metabolic rates and fat stores increase throughout the body including abdominal and, to a lesser extent, hepatic fat [7]. Hibernating mammals such as the yellow-bellied marmot (*Marmota flaviventris*) and 13-lined ground squirrel (*Ictidomys tridecemlineatus*) also become insulin resistant [7,8]. Through a triggering mechanism that remains unclear, the animals then initiate fasting and a reduction in metabolism followed by a drop in body temperature and entry into torpor [9,10]. During torpor the animals generate energy primarily by fat oxidation [6]. Hibernating 13-lined squirrels remain in deep torpor for 1 to 2 weeks, with metabolic rates of 1 to 5 percent of the summer active level, accompanied by severe reductions in heart rate (to 5–10 beats/minute), respiration (5 times/minute) and body temperature (to as low as -2.9°C) [11] Animals then rewarm in about 2 hours and remain at ~37°C for approximately 10 hours (interbout arousal) before cooling again into another bout of torpor. This cycle repeats throughout the winter until the animal finally emerges from hibernation in the spring [5].

The overall response to temperature changes in a circannual hibernator involve local adaptations from multiple central and peripheral organs including the skeletal muscle[12], adipose tissue-brown and white-[13,14,15], heart[16], kidney[17] and liver[18,19,20]. In this regard, in both daily torpor and hibernation, there is a general decrease in metabolic rate allowing animals to cope with cold environments and/or limited food. Among these organs, the liver plays a specific role in body adaptation to torpor. For example, previous studies in hibernating mammals indicate an important reduction in liver mitochondrial respiration [19,20], probably as a ways to reduce thermogenesis and induce energy savings.

One of the most important enzymes involved in controlling fat oxidation is adenosine monophosphate-activated protein kinase (AMPK). In response to an increased ratio of AMP to total ATP, AMPK is activated by phosphorylation [21]. Therefore, in states of energy deficiency the ratio between ATP and AMP or ADP levels is decreased, therefore facilitating the activation of AMPK. Activated AMPK, in turn, stimulates catabolic pathways (fat oxidation, glycolysis and glycogenolysis) while simultaneously inhibiting anabolic pathways (fat synthesis, gluconeogenesis and glycogenesis)[21]. During hibernation, tissues require ATP as the energy source to survive starvation. As a consequence, AMP may accumulate thus acting as an AMPK activator to stimulate β-oxidation of fatty acids. Of interest, AMP can also act as a metabolic substrate for AMPD2 (AMPD2) which converts AMP to inosine monophosphate (IMP). IMP is eventually metabolized by different enzymes into downstream products such as uric acid. Recent studies suggest that the specific activation of AMPD2 decreases the availability of AMP for AMPK activation; hence AMPD2 activation may limit the ability of AMPK to become activated, countering its effects [22,23,24,25].

We therefore tested the hypothesis that hepatic AMPD2 and AMPK have opposing patterns of activity during periods of fat accumulation (summer) and fat oxidation (winter hibernation) in 13-lined ground squirrels traversing their yearly cycle of weight gain and loss.

## Methods

### Ethics statement

All Animal experiments were performed according to protocols approved by the University of Colorado Animal Care and Use Committee.

### Animals

Ground squirrels were obtained from the University of Wisconsin 13-lined ground squirrel captive breeding program at Oshkosh. Ground squirrels were housed at the University of Colorado and maintained under 14 h light-10 h dark conditions with cat chow *ad libitum* until late September or early October when they were transferred to a hibernaculum in which temperatures were maintained at 4°C in darkness; food and water were removed after the animals entered torpor until they began to emerge from hibernation in spring. To identify the various stages of hibernation based on body temperature (Tb), each ground squirrel was surgically implanted intraabdominally with a radiotelemeter and a data logger (VM-FH disks, Minimitter, iButton, Embedded Data Systems) [26].

### Tissue Collection

Ground squirrels (n = 3 to 6 for each of the 7 timepoints) were anesthetized with isoflurane, euthanized by cardiac exsanguination, and the liver tissue snap frozen with liquid nitrogen. Livers were excised *en bloc* and samples were used for immunohistochemistry and oil red O staining (from samples embedded in OCT and frozen) or snap frozen for protein and fat determination.

The timepoints were as follows: *Summer active* (SA), obtained in July and early August; *Fall transition* (FT), obtained in September and October; *Interbout Arousal* (IBA), approximately 3 h after reaching Tb of 35–37°C following a period of torpor; *Entrance* (Ent), Tb decreasing to between 27 and 23°C as animals enter a new bout of torpor; *Early Torpor* (ET), Tb of 4°C for less than 10% of previous torpor bout length; *Late Torpor* (LT), Tb 4°C for 80–95% of the time of the previous torpor bout; *Arousing* (Ar), Tb between 7–12°C during spontaneous arousal from torpor; *Spring Active* (Sp), after emergence from hibernation, the ground squirrel was homeothermic for 11–20 days and had resumed eating, but the hibernaculum was still dark and cold (4°C).

### Protein extraction and western blotting

Protein lysates were prepared from livers employing MAP Kinase lysis buffer as previously described [27]. Six animals per condition (SA, FT, IBA, Ent, ET, LT, Ar and Sp) were analyzed by western blot in two sets of three animals. All proteins analyzed were detected using two separate blots (10% acrylamide) according to their different molecular weights. Blot 1 (dilutions employed in TTBS) was employed for detection of FAS (250 Kda), AMPD2 (90 kDa), P-AMPK (65 kDa) and actin (42 kDa) while in blot 2 ACC (250 kDa), ACL (125 kDa), AMPK (65 kDa) and ECH1 (33 kDa) were detected. Blot 2 was subsequently stripped and reprobed for actin to assure equal protein loading in the blot and to normalize protein abundance. Blots depicted correspond with representative western blots obtained for each protein. Antibody to AMPD2 was obtained from Abnova (1:1000) while antibody to ECH1 was obtained from protein tech (1:500). Sample protein content was determined by the BCA protein assay (Pierce). Approximately 50 mg of tissue was homogenized in 350 μl of MAPK lysis buffer containing proteases and phosphatases inhibitors (Roche), samples were kept on ice for 20 minutes, centrifuged at full speed at 4°C and supernatant collected. 40 μg of total protein were loaded per lane for SDS-PAGE (10% w/v) and then transferred to PVDF membranes. Membranes were incubated with primary antibodies and visualized using a horseradish peroxidase secondary antibody and the HRP Immunstar detection kit (Bio-Rad, Hercules, CA). Chemiluminescence was recorded with an Image Station 440CF and results analyzed with the 1D Image Software (Kodak Digital Science, Rochester, NY). Rabbit polyclonal antibody to AMPD2 (1:1000 dilution in TTBS, H00000271) was purchased from Novus (Littleton, CO), antibodies to β-actin (3700), total ACC (3676), total ACL (4332), total AMPK (2532) and phosphorylated AMPK (Thr172, 2535), to LKB1, P-LKB1, CPT1A, AMPKalpha1, AMPKalpha2, were employed at a 1:1000 dilution in TTBS. and obtained from Cell Signaling (Danvers, MA) while the antibody to ECH1 (11305-1-AP) was purchased from Proteintech (Chicago, IL). Secondary antibodies conjugated with horseradish peroxidase were from Cell Signaling.

### AMPD2 activity assay

AMPD2 activity was determined by estimating the production of ammonia by a modification of the method described by Chaney and Marbach[28] from cells collected in a buffer containing 150 mM KCl, 20 mM Tris-HCl, pH 7.5 1mM EDTA, and 1mM dithiothreitol. Briefly, the reaction mixture consisted of 25 mM sodium citrate, pH 6.0, 50 mM potassium chloride, and the indicated concentrations of AMP. The enzyme reaction was initiated by the addition of the enzyme solution and incubated at 37°C for 15 min for all samples collected. In addition, we measured AMPD2 activity for 15 min at the approximate physiological temperature for each animal with depressed Tb, i.e., 25°C for arousal and entering torpor and 4°C for early and late torpor samples, respectively. The reactions were stopped with the addition of the phenol/hypochlorite reagents: Reagent A (100 mM phenol and 0.050 g/L sodium nitroprusside in water) was added, followed by reagent B (125 mM sodium hydroxide, 200 mM dibasic sodium phosphate, and 0.1% sodium hypochlorite in H_2_O). After incubation for 30 min at 25°C, the absorbance of the samples was measured at 625 nm with a spectrophotometer. To determine the absolute specific activity of ammonia production (micromoles ammonia/min), a calibration curve was determined in the range of 5μM to 1 mM of ammonia.

### Liver Oil Red O staining

Liver tissue was embedded in Optimal Cutting Temperature gel (OCT; Sakura Finetek, Torrance, CA) and frozen in liquid nitrogen. Air-dried cryostat tissue sections (8 *μ*m) were dipped in formalin, washed with running tap water, rinsed with 60% isopropanol, stained for lipids with Oil Red O and counterstained with hematoxylin. Oil red O was then extracted from the slides with isopropanol containing 4% Nonidet P-40, transferred to plastic cuvettes, and optical density (OD) was then measured at a wavelength of 520 nm.

### Determination of intrahepatic triglycerides, β-hydroxybutyrate, inosine, uric acid and phosphate

For triglyceride (TG) determination in liver, fat was solubilized by homogenization in 1 ml solution containing 5% nonidet P40 (NP-40) in water, slowly samples were exposed to 80–100°C in a water bath for 5 minutes until the NP-40 became cloudy, then cooled down to room temperature. Samples were then centrifuged for 2 min to remove any insoluble material. Triglyceride determination with the VetAce autoanalyzer consisted in their initial breakdown into fatty acids and glycerol. Glycerol oxidation generates a product that reacts with the probe to produce color at 570 nm. Similarly, uric acid determination is based in the conversion of uric acid to allantoin, hydrogen peroxide (H_2_O_2_) and carbon dioxide by uricase. The H_2_O_2_ then, is determined by its reaction with the probe to generate color at approximately 571 nm. Values obtained were normalized per mg of soluble protein in the lysates. Hepatic β-hydroxybutyrate, inosine and phosphate levels in MAPK lysates were determined by enzymatic kits (K632, K712 and K410, Biovision, Milpitas, CA).

### Determination of IMP by HPLC

Liver samples were prepared as described[29,30]. In brief, squirrel livers were homogenized with 6 volumes of ice-cold 0.6M perchloric acid. Extracts were then centrifuged at 14,000 g for 5 min and the supernatant was neutralized by adding two volumes of 0.5 M tri-N-octylamine in 1,1,2-trichlorotriflouroethane and mixed for 1 min on a vortex mixer. After centrifugation, an aliquot was injected into the HPLC system. The chromatographic separation of IMP was performed using a ZORBAX Eclipse XDB-C18 column with a mean particular size of 5 mm (Agilent Technologies, Santa Clara, CA). The effluent was monitored at 254 nm and peaks were quantified using peak heights and standard solutions of IMP.

### Plasma metabolites

Acetoacetate and 3-hydroyxbutryate (ketones generated during fat oxidation), uric acid and allantoin were identified and quantified in ground squirrel plasma samples by Metabolon by mass spectrometry as described previously [26].

### Data analysis

All data are presented as the mean ± standard deviation (SD). Data graphics and statistical analysis were performed using Instat (version 3.0) and Prism 5 (both Graph Pad Software, San Diego, CA). Data were analyzed using ANOVA followed by the Tukey-Kramer multiple comparison test with α = 0.05. In all cases experiments were performed 3 times with independent replicates. Total data points (n, number of ground squirrels per time point) are identified in Figure legends.

## Results

### Increased hepatic lipogenesis and development of hepatic steatosis in summer active 13-lined ground squirrels

**Fig 1:**
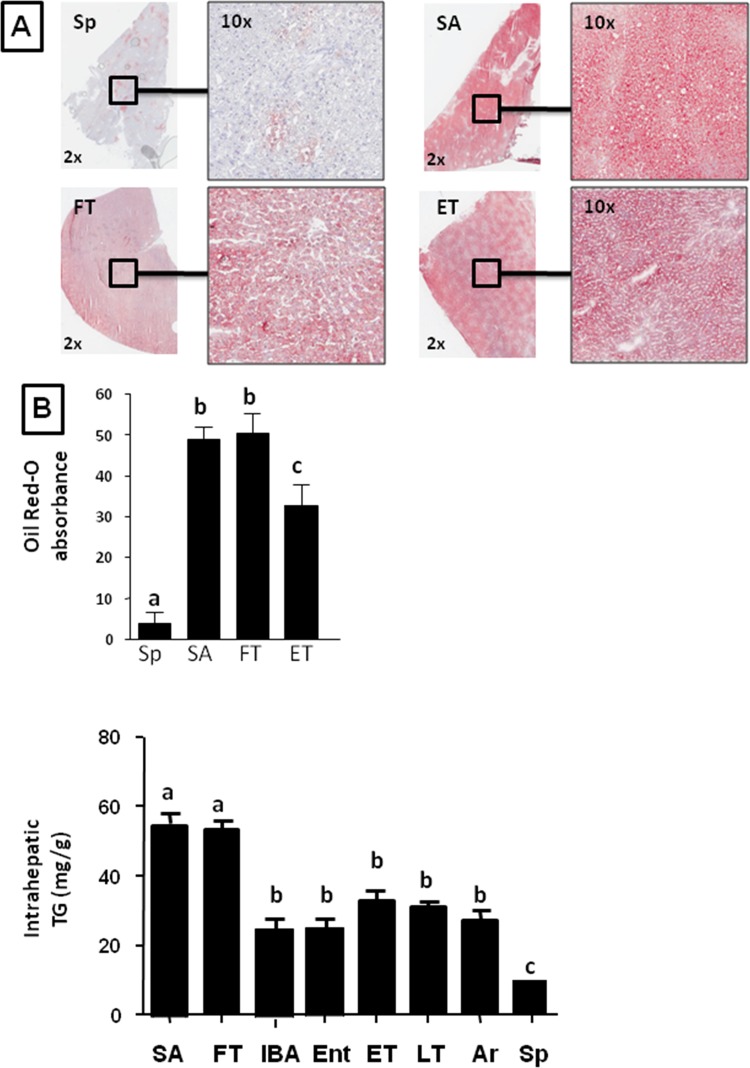
13-lined ground squirrels develop hepatic steatosis when active in summer. (A) Representative images of neutral lipid staining (oil red-O) in livers from animals in spring, summer, early torpor and fall transition. B) Oil red-O quantitation from livers from spring, summer, fall transition, and early torpor animals demonstrate significant increase in hepatic lipid accumulation in summer time that remains high until torpor. C) Hepatic TG quantitation from livers of ground squirrels in summer active (SA), fall transition (FT), interbout arousal (IBA), entering torpor (Ent), early torpor (ET), late torpor (LT), arousing from torpor (Ar), and Spring (Sp) (n≥6 animals per physiological stage, small letters indicate significantly different groups).

**Fig 2:**
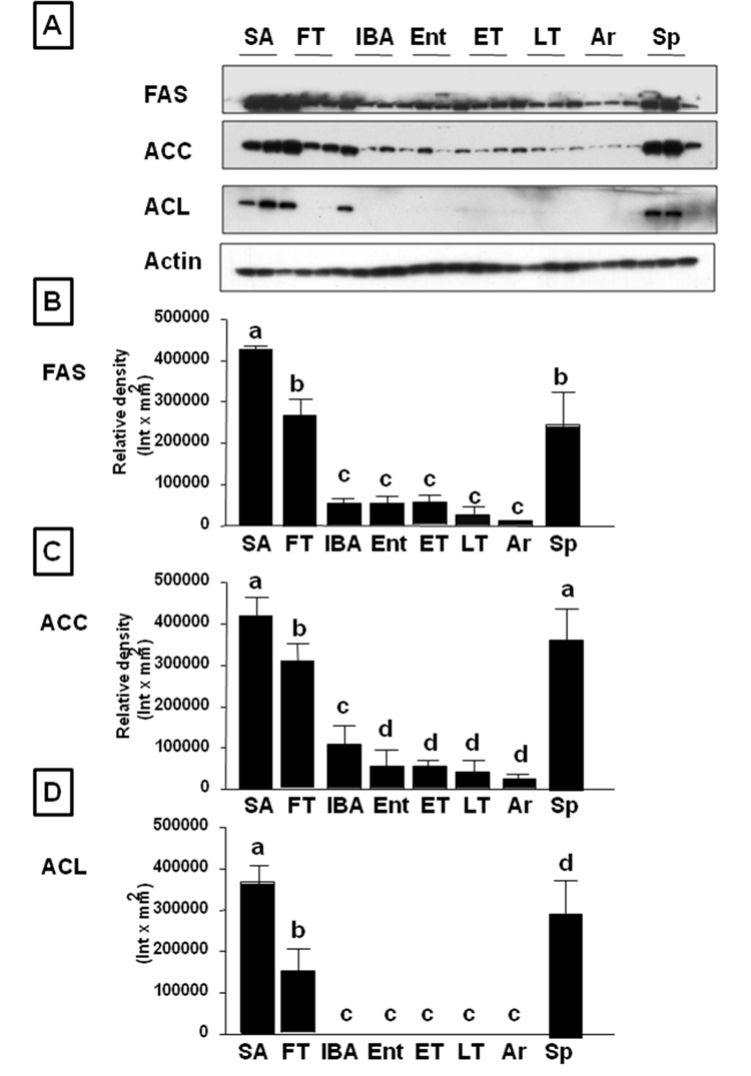
Hepatic lipogenic enzymes are significantly down-regulated as ground squirrels enter into hibernation. A) Representative western blot of lipogenic genes fatty acid synthase (FAS), acetyl-CoA carboxylase (ACC) and ATP-citrate lyase (ACL) in livers from summer active (SA), fall transition (FT), interbout arousal (IBA), entering torpor (Ent), early torpor (ET), late torpor (LT), arousing from torpor (Ar), and Spring (Sp) animals. B-D) Western blot densitometry from all ground squirrels analyzed, n≥6 animals per physiological stage, small letters indicate significantly different groups.

We first evaluated the ground squirrel livers for fat by the presence of oil red-O staining and triglyceride content (Fig 1A–1C). These studies showed that fatty liver (hepatic steatosis) was present in the animals during the summer and fall, and decreased markedly during the hibernation period, with the lowest levels in the spring. During hibernation we could not observe significant differences between different stages although we found a tendency to be lower in IBA and Entering torpor compared with the rest of groups. We also determined the hepatic abundance of various enzymes involved in fat synthesis by western blotting, including fatty acid synthase (FAS), acetyl CoA carboxylase (ACC) and ATP citrate lyase (ACL). All of these enzymes were highest during summer, began to decrease during the fall transition period, were low throughout winter hibernation, and then increased again in the spring (Fig 2).

### Fat oxidation and AMPK are activated in livers of ground squirrels entering hibernation

**Fig 3:**
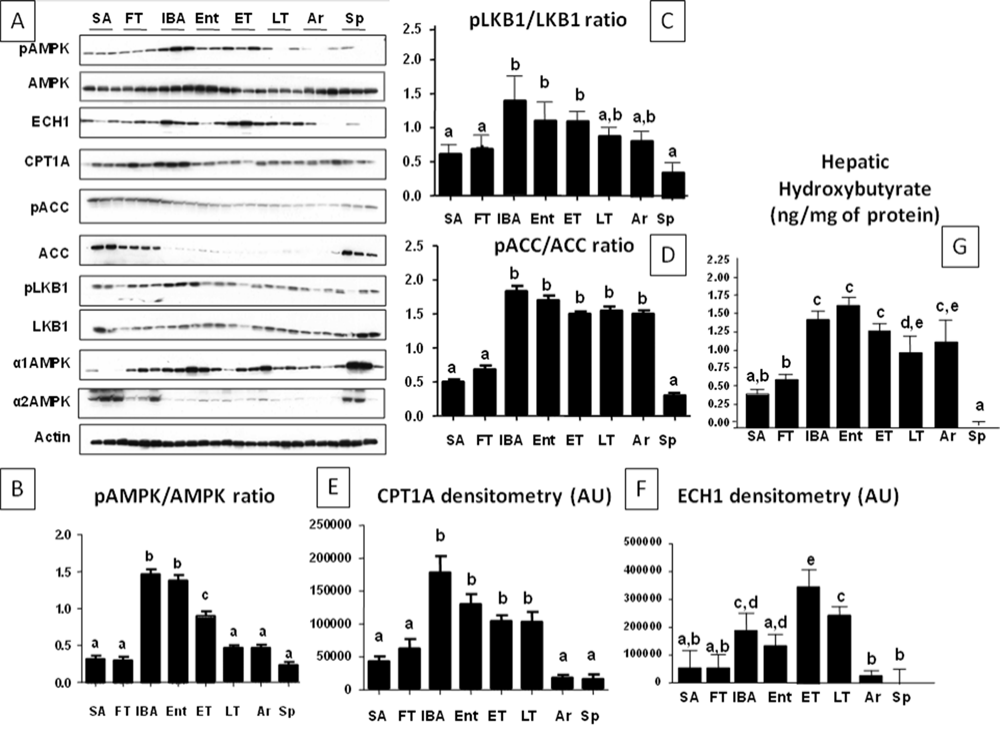
Fat oxidation is activated in liver during hibernation. A) Representative western blot of activated and total AMP-activated protein kinase (P-AMPK, AMPK), inactivated and total ACC (P-ACC, ACC), phosphorylated and total LKB1 (P-LKB1 and LKB1), alpha1 and alpha2 subunits of AMPK, carnitine palmitoyltransferase 1A (CPT1A), and enoyl-CoA hydratase 1 (ECH1) in livers from animals in eight circannual stages (see Fig 2 legend). B-F) Western blot densitometry and P-AMPK/AMPK, P-ACC/ACC ratios from all ground squirrels analyzed. G) Intrahepatic β-hydroxybutyrate (a marker of fat oxidation) levels from all ground squirrels analyzed, n≥6 animals per physiological stage, small letters indicate significantly different groups).

We also examined the abundance of both total and activated AMP kinase. The active AMPK, phosphorylated at threonine 172 (P-AMPK), is important in oxidation of fatty acids. ECH1 (enoyl CoA hydratase-1) and CPT1A (carnitine palmitoyiltransferase 1A)-rate-limiting enzymes in β-fatty acid oxidation- and β-hydroxybutyrate (a ketone generated during fat oxidation) levels were also assessed for a more complete picture of fatty acid oxidation activity. As shown in Fig 3A–3F, activated (phosphorylated) AMPK was low in summer, in association with low levels of ECH1, CPT1A and β-hydroxybutyrate levels, providing evidence that fat oxidation is minimal at this time. This is consistent with the observation that hepatic triglyceride levels are the highest in summer and fall transition (Fig 1C). During the winter torpor-arousal cycle, however, an interesting pattern emerged. Activated P-AMPK was maximally increased during the interbout arousal when the animal had warmed to 37°C. As animals entered into torpor the P-AMPK level began to fall, and by late torpor had reached its lowest winter level, where it remained until the animal warmed again to 37°C (IBA). Total AMPK also reflected a similar pattern as P-AMPK with a predominant expression of the alpha1 subunit (α1) over the alpha2 (α2) which was observed only in summer, fall and spring stages. It is important to note that total AMPK expression began to increase in spring (Sp) even when P-AMPK remained low (Fig 3A). Of interest, P-AMPK expression correlates with the phosphorylative state of ACC. P-ACC (inactive) to ACC ratio, as shown in Fig 3D, is low in summer, fall and spring indicating that liver metabolism is directed toward lipogenesis in these stages. In contrast, during hibernation, P-ACC levels peak at entering and interbout arousal corresponding with the highest P-AMPK to AMPK ratio suggestive of ACC phosphorylation by AMPK. One particular difference is observed at arousal and late torpor in which P-ACC levels remain high despite P-AMPK levels being low. This would suggest that either ACC can be phosphorylated by an AMPK-independent mechanism or that the phosphorylation at ACC is more stable than the one on AMPK. We hypothesize that the mechanism whereby AMPD2 and AMPK counter-regulate is mediated by the pool of AMP available to stimulate each enzyme. AMP is known to activate AMPK allosterically or through liver kinase B1 (LKB1)[31]. As shown in Fig 3, total expression of LKB1 does not substantially change at any stage. However, we found that LKB1 was phosphorylated at serine 334 (P-LKB1) in a pattern similar to the one found for P-AMPK thus suggesting the possibility that LKB1 could be an important mediator in AMPK activation during arousal and in interbout arousal stages. ECH1 and CPT1a expressions increased in winter compared to summer, fall transition and spring. However, within the winter cycle, both ECH1 and CPT1A were significantly decreased at arousal compared to the other stages. β-hydroxybutyrate, reflecting fatty acid oxidation that is controlled by P-AMPK, remained high throughout the winter hibernation stages. Consistently, triglyceride levels at IBA and entering were found to be the lowest among the hibernation states (Fig 1C). The overall pattern suggests that the activation of P-AMPK is dependent on warming to 37°C; this may allow the stimulation of fatty acid oxidation to continue throughout the torpor period.

### AMP Deaminase 2 (AMPD2) Shows an Opposing Activity Pattern Compared to AMP Kinase

**Fig 4:**
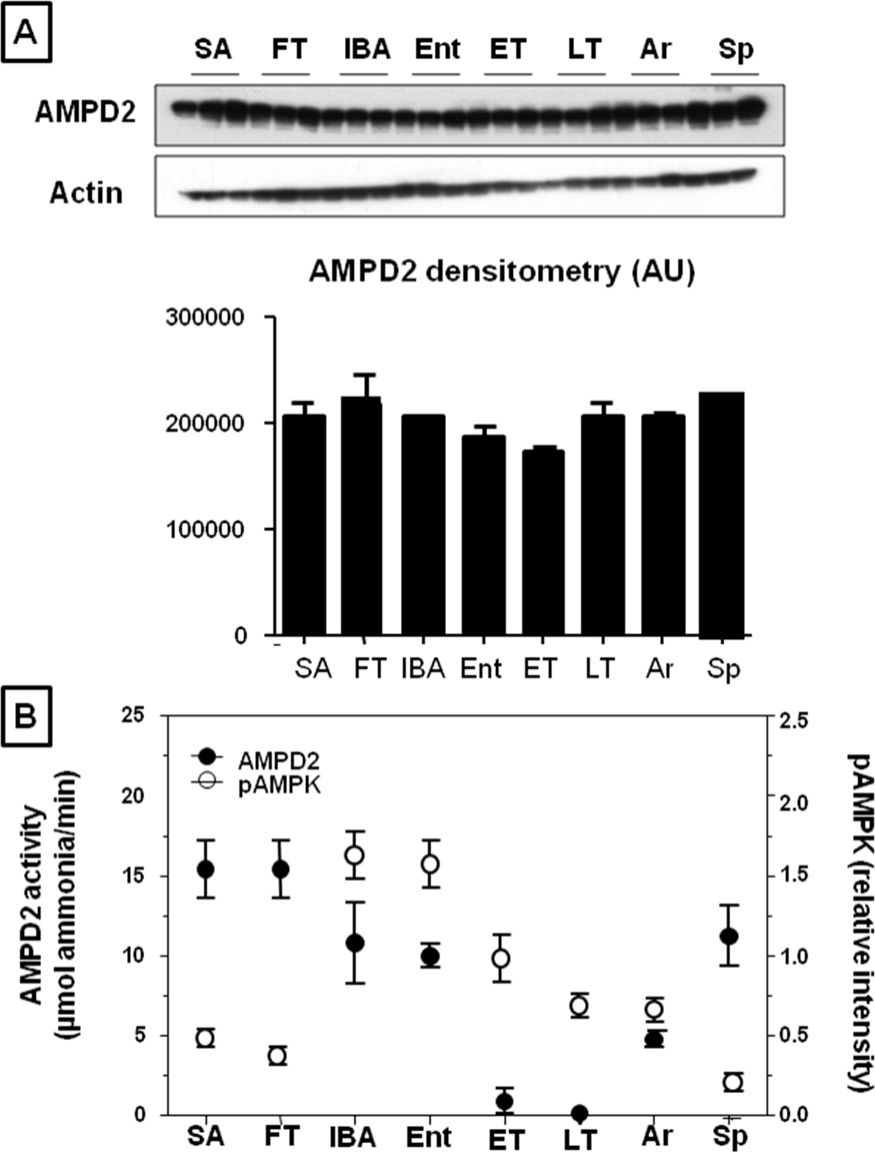
Hepatic abundance and activity of AMPD2 2 (AMPD2) is reduced during hibernation. A) Representative western blot and densitometry of total hepatic AMPD2. B) Plot emphasizing reciprocal relationship between AMPD2 activity and activated P-AMPK (form with phosphorylation at Thr172)/AMPK ratio. AMPD2 activity as measured at the relevant physiological temperature for that state (Table 1, e.g., 4°C for ET and LT, 25°C for Ent and Ar and 37°C for SA, FT, IBA and Sp). Groups are as defined in Fig 2.

AMP activates AMP kinase but is a substrate for degradation by AMPD2 (AMPD2) in the liver. AMPD2 protein abundance tended to be highest in summer (SA) and during the fall transition (FT), with lower levels during the hibernation period, but the differences were not significant (Fig 4A). AMPD2 enzymatic activity was also measured (Fig 4B and Table 1), and found to vary among sample groups when measured at 37°C (ANOVA p = 0.03). Although no significant pairwise differences were identified (Tukey p>0.05), AMPD2 activity tended to be elevated in SA and FT compared to the other groups (Table 1). When the groups with Tb ~37°C (i.e., Sp, SA, FT and IBA) were compared, IBA and Sp were found to have significantly reduced AMPD2 activity compared to SA and FT. Moreover, APMD2 activity was severely reduced at the physiological temperatures of torpor, and had intermediate activity at 25°C (Table 1), consistent with Q_10_ effects (a measure of how changes in temperature affects enzyme activity) and indicating that the enzymatic activity of AMPD2 is immeasurably low while animals are in torpor (ET and LT). Hibernating animals warm briefly to 37°C during each interbout arousal (IBA). The ratio of AMPD2 activity to P-AMPK (reflecting AMPK activity) when the animal’s body temperature is at 37°C (SA, FT, IBA and Sp) is lowest during IBA (ratio 15.71), compared to summer active (SA, ratio 100.25), fall transition (FT ratio 71.42), and spring (Sp ratio 61.11). Thus, the relative activity of AMPD2 to AMPK is higher during the non-hibernating months, declines during the period of hibernation, with marked reduction (to 2% of SA values in LT) at the physiologically relevant temperature of torpor (4°C, Table 1).

**Table 1:** Hepatic AMPD2 activity at different temperatures of hibernation and relationship to activated AMPK.

State	AMPD activity (37°C) (μmol ammonia/min)	AMPD activity (25°C) (μmol ammonia/min)	AMPD activity (4°C) (μmol ammonia/min)	Physiological Temp. AMPD/pAMPK Ratio	37°C AMPD/pAMPK Ratio
**SA**	**15.26±2.6**			100.25(37°C)	100.25
**FT**	**15.39±2.4**			71.42(37°C)	71.42
**IBA**	**10.86±3.1**			15.71(37°C)	15.71
**Ent**	10.56**±4.6**	**10.02±0.2**		16.81(25°C)	17.71
**ET**	11.23**±3.6**		**1.11±0.5**	3.04(4°C)	30.76
**LT**	11.56**±6.2**		**0.55±0.1**	2.04(4°C)	42.97
**Ar**	10.86**±2.2**	**4.95±0.2**		22.29(25°C)	48.91
**Sp**	**10.23±2.0**			61.11(37°C)	61.11

AMPD2 activity was measured in all samples at 37°C and at other physiologically relevant temperatures as indicated (25°C for Ent and Ar, 4°C for ET and LT). The ratio of AMPD2 to activated AMPK was also calculated for both the physiologically relevant temperature and for 37°C

### Changes in Hepatic Uric Acid, inosine, IMP and Phosphate Levels

**Fig 5:**
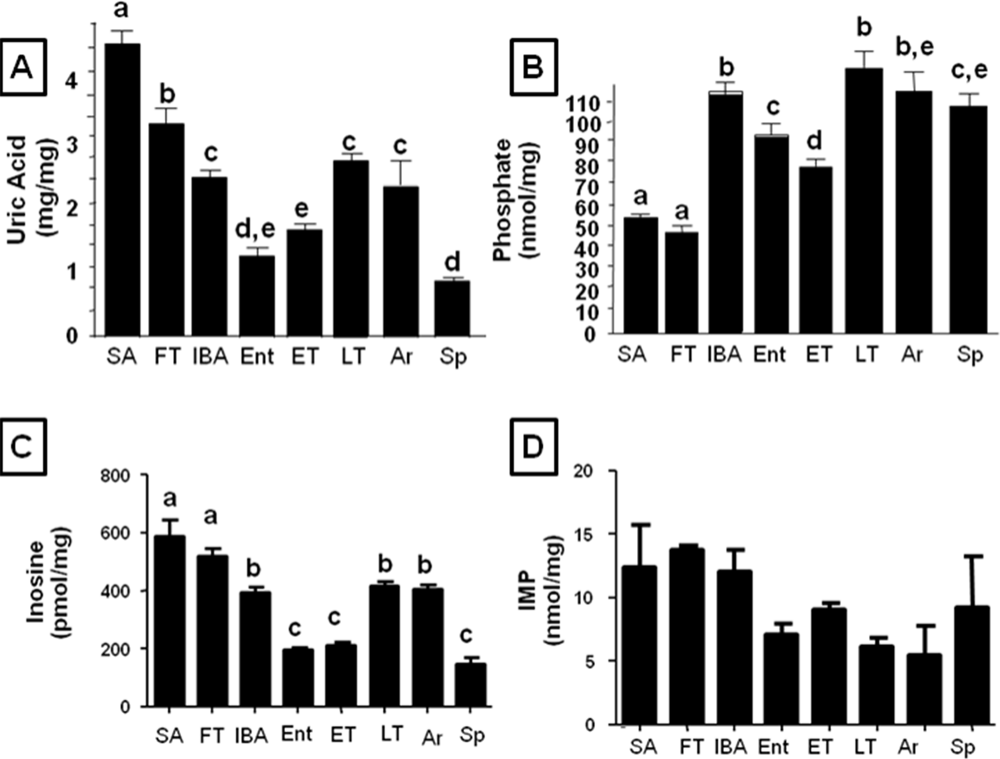
Lower intrahepatic phosphate and higher uric acid levels mirror summer activation of AMPK A) Intrahepatic uric acid) levels in summer active (SA), fall transition (FT), interbout arousal (IBA), entering torpor (Ent), early torpor (ET), late torpor (LT), arousing from torpor (Ar), and Spring (Sp). B) Intrahepatic phosphate (an inhibitor of AMPD2 activity) levels in livers from ground squirrels in summer active (SA), fall transition (FT), interbout arousal (IBA), entering torpor (Ent), early torpor (ET), late torpor (LT) and Spring (Sp). C) Intrahepatic inosine levels in summer active (SA), fall transition (FT), interbout arousal (IBA), entering torpor (Ent), early torpor (ET), late torpor (LT), arousing from torpor (Ar), and Spring (Sp). n≥6 animals per physiological stage, small letters indicate significantly different groups). D) Intrahepatic IMP levels in summer active (SA), fall transition (FT), interbout arousal (IBA), entering torpor (Ent), early torpor (ET), late torpor (LT), arousing from torpor (Ar), and Spring (Sp). n = 3

Inosine and uric acid are products from AMP metabolism through AMPD2. In this regard, in states where AMPD2 is active, levels of uric acid and inosine significantly raise. Consistently, hepatic uric acid, inosine and IMP levels were highest in the summer and fall transition and decreased during the hibernation season (Fig 5A, 5C and 5D). Uric acid accumulated in the liver during torpor (notice the increase between ET and LT), but by the end of the interbout arousal period (IBA), the levels were lowest as animals began to re-enter into the torpid state (Ent). Since AMPD2 activity is known to be mainly inhibited by phosphate [32], we measured hepatic phosphate levels and found that in the summer and fall transition period, intrahepatic free phosphate levels were significantly lower than during hibernation (Fig 5B), consistent with the higher hepatic AMPD2 activity and hepatic uric acid levels observed. The high phosphate levels in the Sp animals may account for the low AMPD2 activity in this group as well.

### Changes in Plasma Metabolites associated with Fat Oxidation

**Fig 6:**
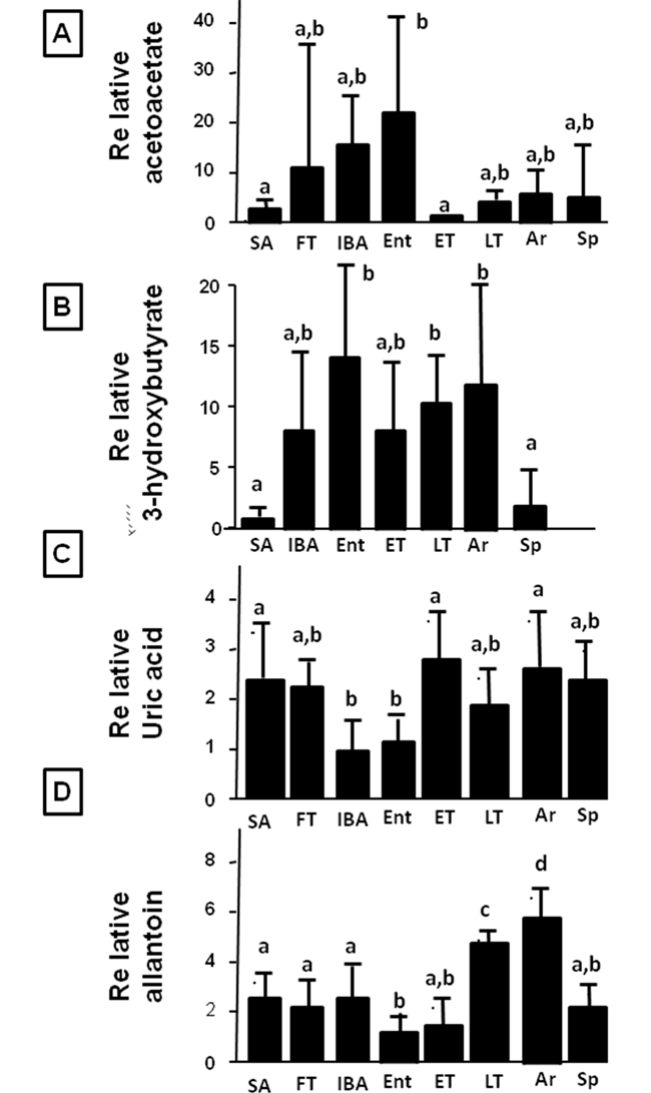
Plasma metabolites acetoacetate, hydroxybutyrate, uric acid and allantoin during hibernation. Fat oxidation markers acetoacetate and hydroxybutyrate (3hydroxybutyrate) (stimulated by activated AMPK) are elevated as animals enter into torpor and levels remain high until spring (A-B). In contrast, uric acid and allantoin levels, markers of AMP catabolism through AMPD2, are elevated in summer with lower levels in torpor. Within the torpor cycle, uric acid levels are being build up during torpor, with lower levels during IBA and entering torpor, probably due to higher uricase activity. P<0.05 (n≥6 animals per physiological stage, small letters indicate significantly different groups).

We also measured plasma levels of fat oxidation markers (acetoacetate and 3-hydroxybutyrate, Fig 6A and 6B) and plasma uric acid and allantoin levels, products of AMP catabolism through AMPD2 (Fig 6C and 6D). Acetoacetate levels were relatively low in the summer (SA) and highly variable in fall (FT). Levels of 3-hydroxybutyrate were consistently increased throughout the hibernation period, mirroring the liver pattern (Fig 3D). However, acetoacetate showed a slightly different pattern with more intra-winter cycling, in that levels were highest in the IBA and Entering periods, and lowest during early torpor (ET).

An interesting relationship was observed for plasma uric acid and allantoin (its metabolite) during torpor-arousal cycles. Uric acid levels were low in IBA and Entering, but accumulated during torpor and early arousing periods. The decreased serum uric acid during IBA was followed by decreased allantoin in Entering. Allantoin began to reaccumulate by ET and continued to accumulate through LT and early arousing.

## Discussion

Circannual hibernators accumulate fat during the summer and live off their fat stores throughout winter [5,6]. While much of the excessive fat is stored in the abdominal adipose depots, the liver is another prime site for fat storage for many animals [33]. In this study we confirm a general pattern consistent with increased fat synthesis and decreased fat oxidation in the liver of 13-lined ground squirrels during the summer period followed by a reversal of that pattern during hibernation in which fat oxidation is high and fat synthesis is low[34]. In this study we specifically evaluated the changes in two enzymes that are important in controlling fat oxidation, AMPK (which stimulates fat oxidation) and AMPD2 which catabolizes AMP, the activator of AMPK, and therefore may have a counteracting role. The primary finding is that there is evidence of dynamic alteration in the activities of these enzymes that is consistent with the changes in fat oxidation observed.

**Fig 7:**
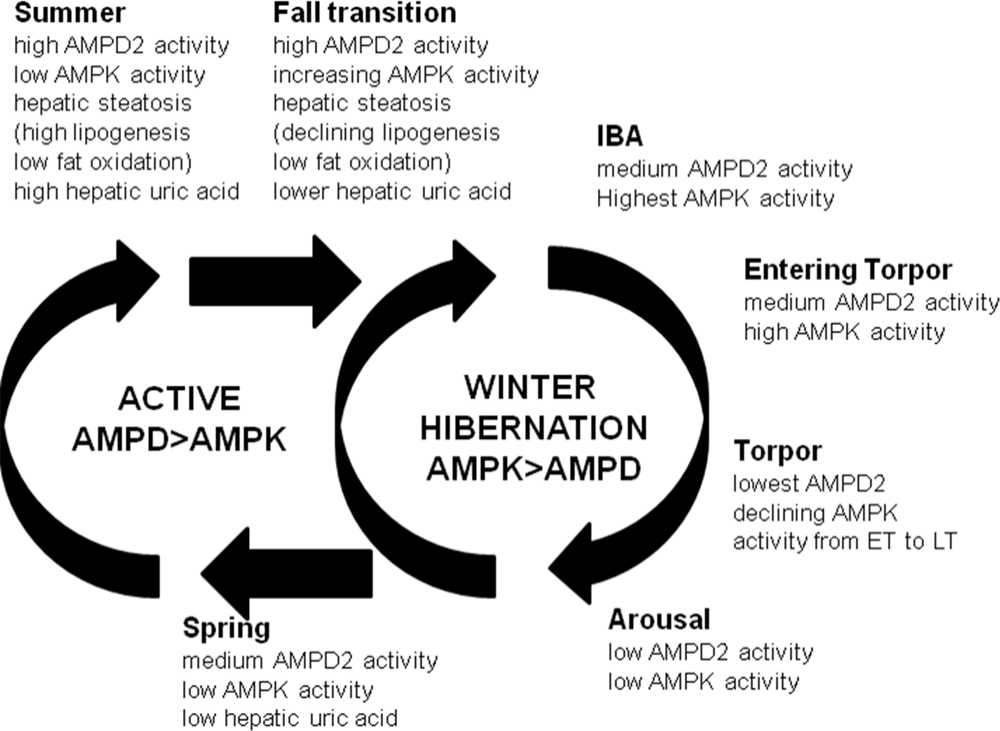
Schematic representation of AMPD2/AMPK switch in the liver of a circannual hibernator. When active in summer, hepatic activity of AMPD2 is elevated along with normal or slightly high uric acid levels and high lipogenic enzymes (FAS, ACC, ACL). In contrast, AMPK activity and ECH1 abundance remain low indicating little or no fat oxidation as reflected by low levels of β-hydroxybutyrate. During fall transition, lipogenic enzyme levels begin to decrease. During hibernation, there is a relative increase in P-AMPK activity to AMPD2 activity during interbout arousals, resulting in the stimulation of fatty acid oxidation that persists during the torpor period despite falling P-AMPK activity. AMPD2 activity is also low during torpor and is associated with a general reduction in intrahepatic uric acid level throughout the hibernation cycle compared to summer. In spring hepatic fat stores are low and fat synthesis returns, in association with a continued inhibition of AMPK activity and a return in AMPD2 activity.

A summary schematic is shown in Fig 7. AMPD2 activity is high during the summer in association with the development of hepatic steatosis and low AMPK activity. During hibernation AMPD2 activity remains modest during the IBA period but falls to very low levels during torpor because of Q10 effects (ET and LT). In contrast, during hibernation animals show a marked increase in AMPK activity, especially at IBA and entering torpor (Ent) states which continues into early torpor (ET) in association with fat oxidation, as suggested by high intrahepatic ECH1, CPT1A and β-hydroxybutyrate levels.

During summer the animal must store fat in preparation for the winter season. We provide evidence for increased fat synthesis with minimal evidence for fat oxidation in the liver in SA animals, in association with the development of moderate hepatic steatosis. During the summer months and fall transition there remains high AMPD2 activity with low levels of activated AMPK in the liver, consistent with a pattern of fat synthesis and storage.

With hibernation a more complex pattern was observed, especially as it relates to AMPK activity and fatty acid oxidation. AMP kinase is well known to be activated during starvation where it has a role in oxidizing fat, and generating ATP [35]; as such, one might expect AMPK to be activated throughout winter hibernation because animals do not eat for several months. Indeed, during hibernation the animal survives by oxidizing fat to generate ATP and other fuels such as β-hydroxybutyrate [36,37,38]. Consistent with this concept, we found that activated AMPK (reflected as P-AMPK) was elevated during several of the winter states, although these high levels were not maintained in LT and Ar. Interestingly, the P-AMPK/AMPK ratio was highest during Ent, but both the enzyme and its activated form decreased as torpor progressed. These observations suggest that AMPK is depleted at the low body temperatures of torpor, but resynthesized during each IBA when the animal’s body temperature is at 37°C, consistent with the known properties of hepatic protein synthesis during torpor-arousal cycles [39]. ECH1 and CPT1A were maintained throughout torpor, but fell during Ar, yet high hepatic β-hydroxybutyrate levels were maintained throughout all of the hibernation stages indicating that fat oxidation continues to some extent during torpor. These data raise the possibility that the intermittent warming and arousals that occur throughout hibernation could be important to reactivate the AMPK fat oxidation pathway to enable the animal to continue to utilize its fat stores.

It should be mentioned that there have been two prior studies that examined hepatic AMPK activation in tissues from hibernating ground squirrels [40,41]. One of these found no significant changes in hepatic AMPK [40,41]. Importantly, these studies only investigated the abundance of P-AMPK during a single unspecified time during torpor and compared those values to non-natural controls, likely obscuring the ability to detect the highly dynamic P-AMPK levels that accompany torpor-arousal and seasonal cycles experienced by the hibernator. By examining AMPK both seasonally and across multiple precisely defined stages in animals undergoing natural torpor-arousal cycles, our studies indicate that hepatic P-AMPK does play a significant role in stimulating fat oxidation, particularly during the brief euthermic periods of hibernation (i.e., IBA).

Besides stimulating AMPK, AMP can also act as a substrate for AMPD2, which depletes AMP by converting it to inosine monophosphate (IMP) and eventually to inosine, hypoxanthine, xanthine, uric acid and, in most mammals including ground squirrels, allantoin. As such, AMPD2 may act to block the activation of AMP kinase by reducing the available pool of AMP [42]. In addition, we have recently discovered that uric acid, which is generated by AMPD2, can function as an inhibitor of AMP kinase and can directly stimulate hepatic fat accumulation [25]. This raises the possibility that intrahepatic uric acid levels, driven by activation of AMPD2, might parallel those associated with fat synthesis, and be opposite to those occurring during fat oxidation.

To evaluate this possibility, we evaluated AMPD2 activity and hepatic uric acid levels at various time points throughout the year. During the summer AMPD2 protein levels were high, associated with high AMPD2 activity and high intrahepatic uric acid, inosine and IMP levels, and this was paralleled by increased abundance of enzymes involved in fat synthesis and elevated hepatic triglyceride levels. During this period P-AMPK was low and there was minimal evidence for fatty acid oxidation.

A more complex pattern was observed during torpor-arousal cycles in hibernation. P-AMPK, increased markedly during interbout arousal and then fell continuously throughout the remainder of the torpor-arousal cycle, consistent with slow degradation in torpor coupled with the inability to initiate protein synthesis until body temperature increases during arousal [39]. On the other hand, AMPD2 activity was decreased throughout winter, but profoundly affected by body temperature, such that it was at just 2–3% of its euthermic values at 4°C. By the end of a torpor bout, both AMPD2 activity and P-AMPK were low during torpor. Intrahepatic uric acid levels were generally lower throughout the hibernation period compared to the summer (SA) and fall transition (FT). Levels were lowest when the animals were entering torpor (Ent), with slow re-accumulation during early and late torpor. Levels stayed higher as animals transitioned (Ar) to the interbout arousal period (IBA), only to plummet again as they re-entered torpor. Our data show that at arousal, the hepatic activities of AMPD2 and AMPK cross and thus, no difference was observed between their activities. At this stage, AMPD activity is being activated, probably as a result of increasing temperature and the necessity of uric acid production as a waste product. As a result of increased AMPD2 activity at arousal, AMP levels drop decreasing AMPK activation. While speculative, the data would be consistent with an initial reduction in uric acid production due to both the lack of energy (and protein) intake during hibernation coupled with a possible mild reduction in AMPD activity. Nevertheless, some uric acid continues to accumulate, even during torpor, likely due in part to the absent renal function during this period [43]. With the interbout arousal, the restoration of brief renal function would allow the excretion of some of the uric acid, facilitating the activation of AMPK.

Previous studies have reported how metabolic rate is modified during hibernation and torpor in mammals[18,19,20]. Metabolic rate drops during torpor with a significant increase at arousal peaking at interbout arousal. The rapid activation of mitochondrial respiration and metabolic rate at arousal and IBA is thought to be mediated by an acute increase in mitochondrial functionality with rapid steps of phosphorylation and deacetylation[19]. Of interest, peak metabolic rate in arousal and IBA correlate with maximal activation of AMPK suggesting that activation of AMPK may be an important step in mitochondrial respiration during torpor. Consistent with this, AMPK phosphorylates and stiimulates the acetylation of multiple mitoochondrial and target genes directed to improve fat and glucose oxidation as well as to increase mitochondrial biogenesis while eliminating defective mitochondria [44]. In this regard, AMPK phosphorylates and inactivates ACC1 and ACC2 to block lipogenesis and stimulate fat oxidation. It also rapidly phosphorylates and inactivates glycogen synthase to shift glucose into glycolysis and oxidative phosphorylation and prevent glycogen accumulation[45]. More importantly, AMPK stimulates the phosphorylation of TORC2 [24] that leads to the deacetylation and activation of PGC1α for de novo mitochondrial biogenesis. It is therefore possible that AMPK activation in torpor is an important step in controlling metabolic rate by modifying phosphorylation and deacetylation states of mitochondrial proteins.

We are unaware of any previous studies that have examined AMPD2 activity in the liver during hibernation. Nevertheless, it is known that both inosine [46] and uric acid levels [46,47], which are both downstream products of AMPD2, are low in the liver of hibernating ground squirrels.

We also examined plasma uric acid and allantoin, the latter being a metabolite of uric acid. Previous studies have found a fall in serum uric acid in the hedgehog when they enter into torpor [48], and likewise with the emergence from hibernation serum uric acid levels rise in several species of hibernating mammals [47,49,50]. Consistent with these observations, plasma uric acid was lowest in IBA and Ent hibernators compared to the other states, Allantoin accumulated during LT and Ar, but dropped to its lowest levels during Ent, both diet and metabolic effects that occur In hibernation states with warm Tb (IBA and Ent) uric acid levels fall, likelydue to decreased protein intake and a mild reduction in AMPD activity, but uric acid slowly accumulates during torpor due to impairment in kidney function. With IBA renal function improves and uricase becomes active, allowing serum uric acid to drop followed by allantoin a few hours later when the animal enters torpor (Ent). An interesting finding in the present study is that the abundance of fat synthesizing enzymes decreases during fall transition prior to entering torpor. This may simply reflect a feedback system in which increasing hepatic fat stores signal a reduction in fat synthesis. Another interesting finding is that spring animals show evidence for minimal fat oxidation, as reflected by low P-AMPK, and low ECH1, CPT1A and β-hydroxybutyrate levels. During this time hepatic triglyceride and uric acid stores are low, but the animals are now actively eating and have switched into a mode to accumulate fat, as reflected by increased FAS, ACC and ACL.

In summary, the hibernating 13-lined ground squirrel actively increases its fat stores in the liver during the summer, and this is associated with relatively high AMPD2 activity, high intrahepatic fat and uric acid stores, and low P-AMPK activity. During hibernation fat synthesis is turned off while fat oxidation is increased and P-AMPK is activated, especially during the IBA. Meanwhile AMPD2 activity remains the same or falls slightly and intrahepatic uric acid levels fall. During torpor both P-AMPK and AMPD2 decline, but some evidence for fat oxidation is still present, likely due to persistent effects of P-AMPK on ECH1 and CPT1A. In the spring the liver fat is nearly absent, and fat oxidation is again turned off and fat synthesis stimulated. During this time intrahepatic uric acid levels are still low. However, while we could not demonstrate this, others have reported that serum uric acid levels increase with emergence from hibernation in spring [47,49,50] consistent with an increase in AMPD2 activity and uric acid production at that time. This study reveals for the first time oscillations in activated AMPK and AMPD associated with the dramatic phenotypic cycles of hibernation. The data indicate a strong association between these patterns and alterations between fat synthesis and fat oxidation, the exact mechanism of which will be an important area for further investigation

## References

[1] Keesey RE, Hirvonen MD Body weight set-points: determination and adjustment. J Nutr. 1997, 127: 1875S–1883S. 9278574 10.1093/jn/127.9.1875S

[2] Bairlein F How to get fat: nutritional mechanisms of seasonal fat accumulation in migratory songbirds. Naturwissenschaften. 2002, 89: 1–10. 12008967 10.1007/s00114-001-0279-6

[3] Robin JP, Boucontet L, Chillet P, Groscolas R Behavioral changes in fasting emperor penguins: evidence for a "refeeding signal" linked to a metabolic shift. Am J Physiol. 1998, 274: R746–753. 9530242 10.1152/ajpregu.1998.274.3.R746

[4] Florant GL, Healy JE The regulation of food intake in mammalian hibernators: a review. J Comp physiol B.2012, 182: 451–467. 10.1007/s00360-011-0630-y 22080368

[5] Carey HV, Andrews MT, Martin SL Mammalian hibernation: cellular and molecular responses to depressed metabolism and low temperature. Physiol Rev. 2003, 83: 1153–1181. 14506303 10.1152/physrev.00008.2003

[6] Dark J Annual lipid cycles in hibernators: integration of physiology and behavior. Annu Rev Nutr. 2005, 25: 469–497. 16011475 10.1146/annurev.nutr.25.050304.092514

[7] Martin SL Mammalian hibernation: a naturally reversible model for insulin resistance in man? Diab Vasc Dis Res. 2008, 5: 76–81. 10.3132/dvdr.2008.013 18537093

[8] Florant GL, Lawrence AK, Williams K, Bauman WA Seasonal changes in pancreatic B-cell function in euthermic yellow-bellied marmots. Am J Physiol. 1985, 249: R159–165. 3895984 10.1152/ajpregu.1985.249.2.R159

[9] Ortmann S, Heldmaier G Regulation of body temperature andenergy requirements of hibernating alpine marmots (Marmota mamota). Am J Physiol Regul Integr Comp Physiol. 2000, 278: R698–R704. 10712291 10.1152/ajpregu.2000.278.3.R698

[10] Sheriff MJ, Williams CT, Kenagy GJ, Buck CL, Barnes BM Thermoregulatory changes anticipate hibernation onset by 45 days: data from free-living arctic ground squirrels. J Comp Physiol B. 2012, 182: 841–847. 10.1007/s00360-012-0661-z 22526260

[11] Barnes BM Freeze avoidance in a mammal: body temperatures below 0 degree C in an Arctic hibernator. Science. 1989, 244: 1593–1595. 2740905 10.1126/science.2740905

[12] Hindle AG, Otis JP, Epperson LE, Hornberger TA, Goodman CA, et al Prioritization of skeletal muscle growth for emergence from hibernation. J Exp Biol. 2015, 218: 276–284. 10.1242/jeb.109512 25452506 PMC4302166

[13] Rouble AN, Tessier SN, Storey KB Characterization of adipocyte stress response pathways during hibernation in thirteen-lined ground squirrels. Mol Cell Biochem. 2014, 393: 271–282. 10.1007/s11010-014-2070-y 24777704

[14] Valente A, Jamurtas AZ, Koutedakis Y, Flouris AD Molecular pathways linking non-shivering thermogenesis and obesity: focusing on brown adipose tissue development. Biol Rev Camb Philos Soc. 2015, 90: 77–88. 10.1111/brv.12099 24708171

[15] Hampton M, Melvin RG, Andrews MT Transcriptomic analysis of brown adipose tissue across the physiological extremes of natural hibernation. PLoS One. 2013, 8: e85157 10.1371/journal.pone.0085157 24386461 PMC3875542

[16] Horwitz BA, Chau SM, Hamilton JS, Song C, Gorgone J, et al Temporal relationships of blood pressure, heart rate, baroreflex function, and body temperature change over a hibernation bout in Syrian hamsters. Am J Physiol Regul Integr Comp Physiol. 2013, 305: R759–768. 10.1152/ajpregu.00450.2012 23904107 PMC3798792

[17] Jani A, Martin SL, Jain S, Keys D, Edelstein CL Renal adaptation during hibernation. Am J Physiol Renal Physiol. 2013, 305: F1521–1532. 10.1152/ajprenal.00675.2012 24049148 PMC4073900

[18] Brown JC, Staples JF Mitochondrial metabolism during fasting-induced daily torpor in mice. Biochim Biophys Acta. 2010, 1797: 476–486. 10.1016/j.bbabio.2010.01.009 20080074

[19] Staples JF Metabolic suppression in mammalian hibernation: the role of mitochondria. J Exp Biol. 2014, 217: 2032–2036. 10.1242/jeb.092973 24920833

[20] Staples JF, Brown JC Mitochondrial metabolism in hibernation and daily torpor: a review. J Comp Physiol B. 2008, 178: 811–827. 10.1007/s00360-008-0282-8 18551297

[21] Hardie DG, Ross FA, Hawley SA AMPK: a nutrient and energy sensor that maintains energy homeostasis. Nat Rev Mol Cell Biol. 2012, 13: 251–262. 10.1038/nrm3311 22436748 PMC5726489

[22] Ouyang J, Parakhia RA, Ochs RS Metformin activates AMP kinase through inhibition of AMP deaminase. J Biol Chem. 2011, 286: 1–11. 10.1074/jbc.M110.121806 21059655 PMC3012963

[23] Plaideau C, Liu J, Hartleib-Geschwindner J, Bastin-Coyette L, Bontemps F, et al Overexpression of AMP-metabolizing enzymes controls adenine nucleotide levels and AMPK activation in HEK293T cells. 2012, FASEB J 26: 2685–2694. 10.1096/fj.11-198168 22415305

[24] Cicerchi C, Li N, Kratzer J, Garcia G, Roncal-Jimenez CA, et al Uric acid-dependent inhibition of AMP kinase induces hepatic glucose production in diabetes and starvation: evolutionary implications of the uricase loss in hominids. 2014, FASEB J 28: 3339–3350. 10.1096/fj.13-243634 24755741 PMC4101654

[25] Lanaspa MA, Cicerchi C, Garcia G, Li N, Roncal-Jimenez CA, et al Counteracting roles of AMP deaminase and AMP kinase in the development of fatty liver.2012, PLoS One 7: e48801 10.1371/journal.pone.0048801 23152807 PMC3494720

[26] Epperson LE, Karimpour-Fard A, Hunter LE, Martin SL Metabolic cycles in a circannual hibernator. Physiol Genomics. 2011, 43: 799–807. 10.1152/physiolgenomics.00028.2011 21540299 PMC3132838

[27] Lanaspa MA, Andres-Hernando A, Rivard CJ, Dai Y, Li N, et al ZAC1 is up-regulated by hypertonicity and decreases sorbitol dehydrogenase expression, allowing accumulation of sorbitol in kidney cells. J Biol Chem. 2009, 284: 19974–19981. 10.1074/jbc.M109.001792 19423711 PMC2740423

[28] Chaney AL, Marbach EP Modified reagents for determination of urea and ammonia. Clin Chem.1962, 8: 130–132. 13878063

[29] Shaw NM, Brown EG, Newton RP Analysis by high-pressure liquid chromatography of the free nucleotide pools of rat tissues [proceedings]. Biochem Soc Trans. 1979, 7: 1250–1251. 535649 10.1042/bst0071250

[30] Smolenski RT, Lachno DR, Ledingham SJ, Yacoub MH Determination of sixteen nucleotides, nucleosides and bases using high-performance liquid chromatography and its application to the study of purine metabolism in hearts for transplantation. J Chromatogr. 1990, 527: 414–420. 2387888 10.1016/s0378-4347(00)82125-8

[31] Lizcano JM, Goransson O, Toth R, Deak M, Morrice NA, et al LKB1 is a master kinase that activates 13 kinases of the AMPK subfamily, including MARK/PAR-1. EMBO J. 2004, 23: 833–843. 14976552 10.1038/sj.emboj.7600110 PMC381014

[32] Lushchak VI, Husak VV, Storey KB Regulation of AMP-deaminase activity from white muscle of common carp Cyprinus carpio. Comp Biochem Physiol B Biochem Mol Biol. 2008, 149: 362–369. 18060819 10.1016/j.cbpb.2007.10.008

[33] Johnson RJ, Stenvinkel P, Martin SL, Jani A, Sanchez-Lozada LG, et al Redefining metabolic syndrome as a fat storage condition based on studies of comparative physiology. Obesity (Silver Spring). 2013, 21: 659–664. 10.1002/oby.20026 23401356 PMC3660463

[34] Hindle AG, Grabek KR, Epperson LE, Karimpour-Fard A, Martin SL (2014) The liver proteome in hibernating ground squirrels is dominated by metabolic changes associated with the long winter fast. Physiol Genomics 2014, 46:10;348–361. 24642758 10.1152/physiolgenomics.00190.2013 PMC4042184

[35] Hardie DG AMP-activated protein kinase: an energy sensor that regulates all aspects of cell function. Genes Dev. 2011, 25: 1895–1908. 10.1101/gad.17420111 21937710 PMC3185962

[36] Lee CC Is human hibernation possible? Annu Rev Med. 2008, 59: 177–186. 10.1146/annurev.med.59.061506.110403 18186703

[37] Andrews MT, Russeth KP, Drewes LR, Henry PG Adaptive mechanisms regulate preferred utilization of ketones in the heart and brain of a hibernating mammal during arousal from torpor. Am J Physiol Regul Integr Comp Physiol. 2009, 296: R383–393. 10.1152/ajpregu.90795.2008 19052316 PMC2643978

[38] Al-Badry KS, Taha HM Hibernation-hypothermia and metabolism in hedgehogs. Changes in water and electrolytes. Comp Biochem Physiol A Comp Physiol. 1083, 74: 435–441. 6131787 10.1016/0300-9629(83)90627-8

[39] van Breukelen F, Martin SL Translational initiation is uncoupled from elongation at 18 degrees C during mammalian hibernation. Am J Physiol Regul Integr Comp Physiol. 2001, 281: R1374–1379. 11641105 10.1152/ajpregu.2001.281.5.R1374

[40] Healy JE, Gearhart CN, Bateman JL, Handa RJ, Florant GL AMPK and ACCchange with fasting and physiological condition in euthermic and hibernating golden-mantled ground squirrels (Callospermophilus lateralis). Comp Biochem Physiol A Mol Integr Physio. 2011,l 159: 322–331. 10.1016/j.cbpa.2011.03.026 21473923 PMC3090470

[41] Horman S, Hussain N, Dilworth SM, Storey KB, Rider MH Evaluation of the role of AMP-activated protein kinase and its downstream targets in mammalian hibernation. Comp Biochem Physiol B Biochem Mol Biol. 2005, 142: 374–382. 16202635 10.1016/j.cbpb.2005.08.010

[42] Plaideau C, Liu J, Hartleib-Geschwindner J, Bastin-Coyette L, Bontemps F, et al Overexpression of AMP-metabolizing enzymes controls adenine nucleotide levels and AMPK activation in HEK293T cells. FASEB J. 2012, 26:6;2685–2694 22415305 10.1096/fj.11-198168

[43] Jani A, Epperson E, Martin J, Pacic A, Ljubanovic D, et al Renal protection from prolonged cold ischemia and warm reperfusion in hibernating squirrels. Transplantation. 2011, 92: 1215–1221. 10.1097/TP.0b013e3182366401 22082817

[44] Mihaylova MM, Shaw RJ The AMPK signalling pathway coordinates cell growth, autophagy and metabolism. Nat Cell Biol. 2011, 13: 1016–1023. 10.1038/ncb2329 21892142 PMC3249400

[45] Skurat AV, Roach PJ (Phosphorylation of sites 3a and 3b (Ser640 and Ser644) in the control of rabbit muscle glycogen synthase. J Biol Chem. 1995, 270: 12491–12497. 7759494 10.1074/jbc.270.21.12491

[46] Nelson CJ, Otis JP, Martin SL, Carey HV Analysis of the hibernation cycle using LC-MS-based metabolomics in ground squirrel liver. Physiol Genomics. 2009, 37: 43–51. 10.1152/physiolgenomics.90323.2008 19106184

[47] Toien O, Drew KL, Chao ML, Rice ME Ascorbate dynamics and oxygen consumption during arousal from hibernation in Arctic ground squirrels. Am J Physiol Regul Integr Comp Physiol. 2001, 281: R572–583. 11448862 10.1152/ajpregu.2001.281.2.R572

[48] Al-Badry KS, Taha HM Hibernation-hypothermia and metabolism in hedgehogs. Changes in some organic components. Comp Biochem Physiol A Comp Physiol. 1983, 74: 143–148. 6130876 10.1016/0300-9629(83)90725-9

[49] Drew KL, Toien O, Rivera PM, Smith MA, Perry G, et al Role of the antioxidant ascorbate in hibernation and warming from hibernation. Comp Biochem Physiol C Toxicol Pharmacol. 2002, 133: 483–492. 12458177 10.1016/s1532-0456(02)00118-7

[50] Okamoto I, Kayano T, Hanaya T, Arai S, Ikeda M, et al Up-regulation of an extracellular superoxide dismutase-like activity in hibernating hamsters subjected to oxidative stress in mid- to late arousal from torpor. Comp Biochem Physiol C Toxicol Pharmacol 2006, 144: 47–56. 16807121 10.1016/j.cbpc.2006.05.003

